# Efficacy of mupirocin, neomycin and octenidine for nasal *Staphylococcus aureus* decolonisation: a retrospective cohort study

**DOI:** 10.1186/s13756-021-01043-1

**Published:** 2022-01-10

**Authors:** J. Allport, R. Choudhury, P. Bruce-Wootton, M. Reed, D. Tate, A. Malviya

**Affiliations:** Northumbria Healthcare NHS Trust, Department of Trauma and Orthopaedics, Wansbeck General Hospital, Woodhorn Lane, Ashington, NE63 9JJ UK

**Keywords:** Methicillin sensitive *Staphylococcus aureus*, Surgical site infection, Periprosthetic joint infection, MSSA, Decolonisation

## Abstract

**Background:**

Periprosthetic joint infection (PJI) causes significant morbidity. Methicillin sensitive *Staphylococcus aureus* (MSSA) is the most frequent organism, and the majority are endogenous. Decolonisation reduces PJIs but there is a paucity of evidence comparing treatments. Aims; compare 3 nasal decolonisation treatments at (1) achieving MSSA decolonisation, (2) preventing PJI.

**Methods:**

Our hospital prospectively collected data on our MSSA decolonisation programme since 2013, including; all MSSA carriers, treatment received, MSSA status at time of surgery and all PJIs. Prior to 2017 MSSA carriers received nasal mupirocin or neomycin, from August 2017 until August 2019 nasal octenidine was used.

**Results:**

During the study period 15,958 primary hip and knee replacements were performed. 3200 (20.1%) were MSSA positive at preoperative screening and received decolonisation treatment, 698 mupirocin, 1210 neomycin and 1221 octenidine. Mupirocin (89.1%) and neomycin (90.9%) were more effective at decolonisation than octenidine (50.0%, *P* < 0.0001). There was no difference in PJI rates (*P* = 0.452).

**Conclusions:**

Mupirocin and neomycin are more effective than octenidine at MSSA decolonisation. There was poor correlation between the MSSA status after treatment (on day of surgery) and PJI rates. Further research is needed to compare alternative MSSA decolonisation treatments.

## Background

Hip and knee arthroplasty offers excellent outcomes for end-stage osteoarthritis and other painful conditions [1]. Periprosthetic joint infection (PJI) causes considerable morbidity and mortality, 5-year mortality rates for hip PJI are upto 21% [2]. Modern advances have reduced the rates of PJI [3] but it continues to represent a significant burden with rates typically around 1% for both hip and knee arthroplasty [4]. In 2019 infection accounted for 12% (875/7080) of hip revisions and 16% (916/5599) knee revisions in the England and Wales joint registry [5]. Associated healthcare costs are as high as £50,000 per case in the UK [6].

Methicillin sensitive *Staphylococcus aureus* (MSSA) is the most common organism isolated in early postoperative PJI [7], accounting for upto 38% of cases [8]. A link between MSSA nasal colonisation and surgical site infections (SSI) has long been established, carriers of MSSA have a 5.9 times higher risk of developing an MSSA PJI [9]. DNA analysis has shown that endogenous nasal flora matches PJI isolates in 80% of cases [7, 10, 11]. Nasal MSSA carriage rates vary depending on population studied, estimates from a range of international studies puts the rate at 20–30% in the general population [7, 11, 12].

There is strong evidence that nasal *S. aureus* decolonisation is effective at reducing PJIs [13, 14]. However, mupirocin nasal ointment is the only treatment with good quality evidence. In a recent meta-analysis all nine studies included used the same eradication treatment [13]. There is very little literature comparing decolonisation agents. With the risks of drug resistance research is needed into alternative therapies to mupirocin [15].

Octenidine HCl is a topical antiseptic with activity against both gram-positive and gram-negative bacteria. As an antiseptic nasal gel octenidine can be supplied by the surgical pre-assessment team without prescription, streamlining the process, and reducing cost. Although it has less potential for inducing resistance than mupirocin [16], there is some reduction in bacterial susceptibility to octenidine HCl emerging [17].

Neomycin is an aminoglycoside antibiotic active against both gram-positive and gram-negative bacteria. There is limited research into the efficacy of neomycin ointment for nasal MSSA decolonisation. Leigh et al. showed neomycin achieved nasal decolonisation in 61% of cases compared to 95% with mupirocin at 8 days after treatment [18]. Resistance to neomycin has been reported as high as 42% in a study from Brazil, the authors note this is likely due to its popular use without prescription in the country [19].

This study aims to compare the efficacy of nasal mupirocin, neomycin or octenidine, as part of an MSSA eradication bundle with octenidine skin wash, at both achieving MSSA decolonisation and reducing rates of MSSA PJI in primary hip or knee replacement.

## Methods

In 2010 our organisation introduced an MSSA screening and decolonisation programme for patients undergoing elective hip and knee joint arthroplasty. Patients underwent seperate groin and nose swabs for detection of MSSA during their preoperative assessment. Liquid swabs (Medical Wire, Corsham, UK) were originally used until a change to flocked swabs (COPAN, Brescia, Italy) in June 2018. Swabs were directly streaked onto Colorex *S. aureus* media (E&O Laboratories Ltd, England) without pre-enrichment. Plates were read at 18 and 24 h, further identification was performed using the VITEK MS (MALDI TOF, Biomerieux, France) and sensitivity testing using the VITEK 2. Clinical and Laboratory Standards Institute (CLSI) breakpoint standards were used to define methicillin sensitivity. Screening was performed around 14 days prior to surgery, if surgery was delayed beyond 28 days then screening was repeated. Patients testing positive for MRSA were excluded from this study.

Patients testing positive for MSSA carriage were given nasal decolonisation treatment to use 5 days prior to surgery and 5 days after (10 days total). Group one, from 2010 until August 2017, used nasal mupirocin 2% (Bactroban, GlaxoSmithKline UK Ltd, Brentford, UK). Group two, from August 2017, used an octenidine HCl 0.1% nasal decolonisation agent (Octenisan Nasal Gel, Schϋlke & Mayr UK Ltd, Sheffield, UK). Group three used neomycin 0.5% and Chlorhexidine 0.1% nasal cream (Naseptin, Alliance Pharmaceuticals Ltd, Chippenham, UK), this was used as an alternative to mupirocin due to periods of national supply shortage. All patients received an octenidine HCl body wash (Octenisan, Schϋlke & Mayr UK Ltd, Sheffield, UK) for daily use 5 days prior to surgery.

Patients received verbal and written instructions on how to apply both the nasal treatment and the body wash. Octenisan body wash was applied, once per day, to wet hair and body using a wash cloth, after 1 min skin contact this was washed off. Hair was only included on alternate days (days two and four). Mupirocin and octenidine were both applied twice daily, neomycin was applied four times per day. The steps for nasal treatment are:

1. Nasal cavity cleared if needed.
2. Cream introduced into the anterior nasal cavity with a finger or cotton bud for 2 min.
3. Cream spread further by pressing the sides of the nose together.
4. Any excess cream removed.

Primary outcome was decolonisation efficacy, measured by MSSA positive culture on the day of surgery. The secondary outcome was MSSA PJI.

**Fig. 1:**
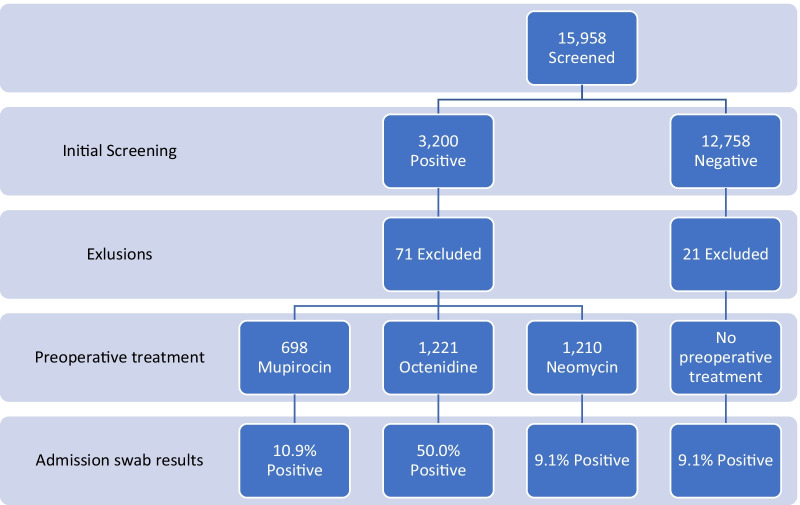
Patient cohort

Data was collected prospectively by the SSI surveillance team locally on all MSSA carriers from September 2013. This data has been retrospectively analysed for this study. Four groups of interest were identified from this database. The first three groups were positive at initial screening and treated as per protocol at the time, receiving nasal mupirocin, octenidine or neomycin. The fourth were negative at initial screening but positive when swabbed on the day of surgery (Fig. 1), this group would have received nasal decolonisation treatment commencing post-operatively for a duration of 5 days. Group four likely represents false negative results from initial screening as well as intermittent carriers.

Microbiology records were reviewed for MSSA status on admission for surgery. Although repeat testing was not specified in the decolonisation protocol the majority were tested. Admission swabs were performed and tested as previously stated for initial screening.

The SSI surveillance team also prospectively record cases of PJI, diagnosed as per Public Health England (PHE) criteria [20]. Cases of PJI within 90 days of surgery were cross referenced with the MSSA carrier database. Cases with less than 90 days follow up were excluded. The causative organism was reviewed for each case, if MSSA was isolated as part of a multi-organism growth this was included as an MSSA PJI.

### Statistical analysis

Statistical analysis was performed using Statistical Package for the Social Sciences (SPSS Version 26). All results quoted to two significant figures. Categorical data was analysed using chi squared test.

## Results

**Fig. 2:**
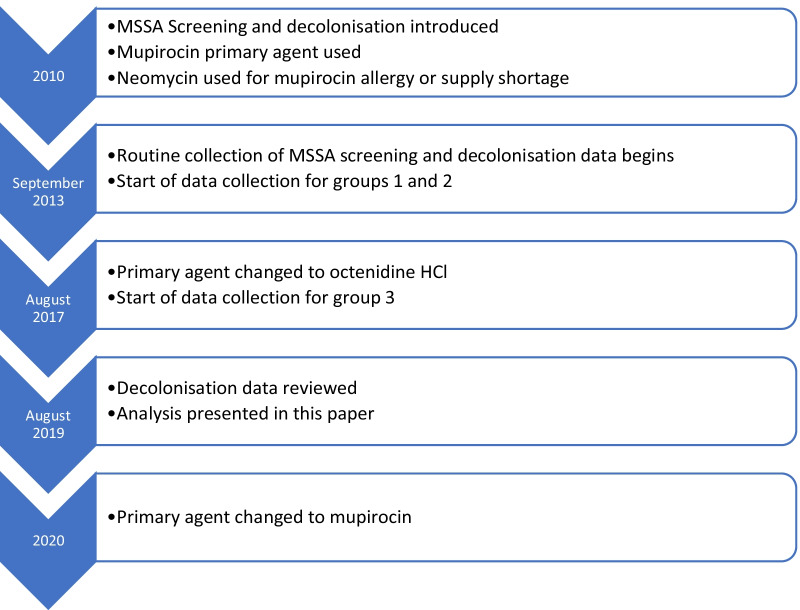
Chronology of different phases studied

Data for cases from September 2013 to August 2019 was analysed. Groups one and two covers a period of 47 months, group three covers 24 months (Fig. 2). During this period 15,958 hip and knee arthroplasty procedures were performed. 3200 (20.1%) MSSA carriers were identified, 71 were excluded as follow up was less than 90 days. 698 received treatment with mupirocin, 1210 received neomycin and 1221 octenidine (Fig. 1). Of the 12,758 negative on initial screening, 1164 (9.1%) were subsequently positive on admission, 21 excluded with less than 90 days follow up (group 4). The combined total of all four groups of MSSA carriers was 4272.

### MSSA decolonisation

Microbiology records were reviewed, in total 1776 patients (56.8%) were swabbed for MSSA on day of surgery (Table 1). The difference in proportion swabbed in each group did reach statistical significance (*P* < 0.001). Significantly more patients remained colonised with MSSA after treatment with octenidine (50.0%) than with mupirocin (10.9%, *P* < 0.00001) or with neomycin (9.1%, *P* < 0.00001). There was no statistical difference between mupirocin and neomycin (*P* = 0.393).

**Table 1:** MSSA swab results on day of surgery

	Group 1 (mupirocin)	Group 2 (octenidine)	Group 3 (neomycin)	Total
Swabbed on day of surgery	322/698 (46.1%)	830/1221 (68.0%)	624/1210 (51.6%)	1776/3129 (56.8%)
Swab positive	35/322 (10.9%)	415/830 (50%)	57/624 (9.1%)	507/1776 (28.5%)
Swab negative	287/322 (89.1%)	415/830 (50%)	567/624 (90.9%)	1269/1776 (72.5%)

### Periprosthetic joint infection

Between the four groups, 29 deep infections were diagnosed within 90 days of the index procedure (0.68% PJI rate), MSSA was isolated from deep tissue samples in ten cases (0.23% MSSA PJI rate). There was no statistical difference between the four groups (Table 2). Causative organisms are presented for information (Table 3). Multi-organism cases are listed as such, one multi-organism case in group three also included MSSA. A further 82 PJI infections were identified amongst patients that were not MSSA carriers, a comparable rate of 0.71%.

**Table 2:** Periprosthetic joint infection rates

	Group 1 (mupirocin)	Group 2 (octenidine)	Group 3 (neomycin)	Group 4	Total	P-Value
MSSA PJI	2/698 (0.29%)	3/1221 (0.25%)	1/1210 (0.08%)	4/1143 (0.35%)	10/4272 (0.23%)	0.452
All PJI	6/698(0.86%)	3/1221 (0.25%)	8/1210 (0.66%)	12/1143 (1.05%)	29/4272 (0.68%)	0.108

**Table 3:** Periprosthetic joint infections—causative organisms

	Group 1 (mupirocin)	Group 2 (octenidine)	Group 3 (neomycin)	Group 4
*Staphylococcus aureus* (MSSA)	2	3	1	3
*Staphylococcus epidermidis*	1	0	2	1
*Escherichia coli*	1	0	2	1
*Proteus mirabilis*	0	0	0	1
*Staphylococcus lugdunensis*	0	0	1	0
*Corynebacterium striatum*	0	0	1	0
*Streptococcus dysgalactiae*	0	0	1	0
*Morganella morganii*	1	0	0	0
Multi-organism	1	0	0	3 (1 included MSSA)
No growth	0	0	0	3

All six cases, in the treatment groups, that developed an MSSA PJI were swabbed on the day of surgery, one was positive for MSSA. Of the cases that developed PJI regardless of organism, 12 out of 17 were swabbed on the day of surgery with one positive.

### Intermittent carriers

Group four includes patients that were negative at pre-assessment screening but positive on day of surgery, this occurred in 1164 cases (1164/12,758, 9.1%). This is likely to represent both false negatives at screening and intermittent carriers.

## Discussion

*Staphylococcus aureus* is recognised as the most common causative organism in early postoperative PJI [7]. Since the majority are associated with an endogenous source [11, 21, 22], MSSA colonisation is considered a modifiable risk factor. A Cochrane review showed decolonisation was effective at reducing nosocomial infections (RR 0.55 [23, 24]). Similarly, a meta-analysis by Zhu et al. concluded MSSA screening and decolonisation significantly reduced PJI (OR 0.40) [13]. Decolonisation forms part of the World Health Organisation SSI guidelines [25].

The topical antibiotic mupirocin is the only treatment that currently has good study data. The majority of existing research focuses on decolonisation as a method to reduce SSI and nosocomial infections i.e. an active treatment is compared to a non-active control. There are very few studies comparing different treatment options. In the UK, National Institute for Clinical Excellence (NICE) guidance states mupirocin should be considered whenever MSSA is a likely cause of SSI [26].

Mupirocin is a topical antibiotic working through inhibition of bacterial isoleucyl-tRNA synthetase [27]. Widespread use of mupirocin for MSSA decolonisation is however leading to the emergence of resistance. High level resistance is associated with the mupA gene whereas chromosome point mutations lead to low level resistance (prevalence 7.6% and 8.5% respectively) [28]. One study in Brazil demonstrated a significant drop in mupirocin resistance when they were able to restrict mupirocin use within their population [29]. Establishing treatments comparable, or superior, to mupirocin is key to combating resistance and further reducing PJI rates. Alternative agents for MSSA decolonisation are already in clinical use. Further treatments are at various stages of development and show promise [7, 15, 30].

In our cohort of 3129 patients, mupirocin and neomycin were significantly more effective at MSSA decolonisation (89% and 91% of patients decolonised respectively) than octenidine (50% decolonised). On the day of surgery patients were 5.1 times more likely to be positive on swab culture when treated with octenidine. In the largest study of nasal decolonisation Perl et al. compared mupirocin to placebo in over 4000 patients [21]. Mupirocin achieved nasal decolonisation rates of 83.4% compared to 27.4% where placebo was used. Perez-Fontan et al. compared mupirocin to neomycin in patients undergoing peritoneal dialysis, they found that 100% were decolonised in the mupirocin arm but only 40% by neomycin immediately after treatment, re-colonisation occurred in both groups [31]. It is however worth noting that they used a 0.1% w/w preparation of neomycin compared to 0.5% w/w in our study. When used for decolonisation in families with recurrent *S. aureus* infections, nasal decolonisation rates were 95% for mupirocin and 61% for neomycin 8 days after treatment [18]. The authors are not aware of any papers presenting decolonisation rates for nasal octenidine for MSSA. However, several studies report that where nasal octenidine is included as part of an MRSA decolonisation protocol, treatment was successful in 68.0–93.3% of patients [32, 33]. Our findings are largely in line with results from current literature.

Despite the significant difference in decolonisation efficacy, in our study, this did not translate into a difference in MSSA PJI rate. There was no statistical difference in MSSA PJI rate between the three treatment groups or with group 4. The authors acknowledge that the sample size is too small to draw firm conclusions on PJI.

Interestingly, all three infected cases in group 3 (octenidine) were caused by MSSA. There were no PJI caused by other organisms. Octenidine has a broad spectrum of activity [34], it may be the case that it is more effective at preventing non-MSSA PJI. There was a trend toward lower overall PJI rates in this group, however this did not reach statistical significance.

A meta-analysis in 2020 showed that mupirocin nasal decolonisation reduced PJI compared to control groups (placebo or no treatment) [13]. Using a before-after cohort study, Reiser et al. investigated the use of nasal octenidine, along with an octenidine body wash, for preventing SSI in cardiothoracic surgery [35]. They concluded that octenidine decolonisation did not reduce SSIs overall, however it did reduce some specific infections (harvest site and organ/space sternal SSIs). To the best of our knowledge no studies have presented SSI or PJI rates with the use of nasal neomycin for decolonisation.

Phillips et al. compared mupirocin to a povidone-iodine solution for nasal decolonisation in a cohort undergoing arthroplasty or spinal fusion [36]. Similar to our study they found that decolonisation efficacy did not necessarily translate into efficacy at preventing SSI. Mupirocin achieved better decolonisation rates 1–3 days after treatment (92% vs 54%) but Povidone-iodine had lower rates of *S. aureus* SSI. Povidone-iodine is of particular interest as it is applied as a single treatment on the day of surgery potentially reducing issues of compliance. MSSA decolonisation is dependent on the total number of doses received [21]. Our institution did not routinely assess patient compliance with treatment. The observed differences between the groups may be related to variations in compliance between the treatments rather than directly related to the efficacy of the treatment. Future prospective research should investigate patient compliance and factors related to each treatment affecting compliance.

The European Medicines Agency (EMA) has recommended that, based on a presumed impact on clinical end points, microbiological outcomes can used when evaluating nasal decolonisation for orthopaedic or cardiac surgery [37]. Our results, along Phillips et al., highlight a discrepancy between decolonisation efficacy and infection rates. It is likely that bacterial load plays a role. Persistent MSSA carriers have been shown to have a higher nasal bacterial load and this translates into a higher risk of infection than intermittent carriers [38, 39]. Treatments may prevent PJI by decreasing the bacterial load, without crossing the threshold of sensitivity for the screening test used. This is particularly true if highly sensitive polymerase chain reaction (PCR) is used for screening [40]. Consideration also has to be given to the possibility of detecting non-viable organisms when PCR is used, or the effect of any decolonisation agent picked up by swabs when using culture plates. Caution should be used when using MSSA nasal eradication as an endpoint in research instead of PJI or SSI.

MSSA carriers represented 20% of our cohort, with a further 9.1% newly positive on day of surgery, possibly representing intermittent carriers. Despite commencing treatment peri-operatively, this 4^th^ group also had no statistical difference in deep infection rates. Bacterial density clearly plays a significant role that is not fully understood but could explain why intermittent carriers and incomplete eradication may not increase the risk of PJI. Around one third of patients with MSSA carriage appear to have intermittent carriage which can be missed on screening. This supports the concept of universal decolonisation for all patients undergoing high risk surgery without screening, or the use of more sensitive detection techniques such as PCR. Previous cost analysis published from our instituition showed screening costs of £8 per patient compared to £7.73 for decolonisation treatment. Universal decolonisation without screening is cheaper (£8 saving for carriers and £0.27 for non-carriers) and avoids missed carriers. The potential downside is the development for increased resistance with increased use of mupirocin. This further highlights the need for research to compare alternative agents.

## Limitations

The main limitation of this study is the lack of randomisation. The data covers 6 years, during which multiple changes were made to standard practice and surgical techniques to improve patient care including reducing infections. The PJI rate did decrease markedly over this time, this is evident in the difference in overall PJI rate between the two treatment groups in Table 1 which approached statistical significance (*P* = 0.108). This reduction could have impacted on the MSSA PJI rates, concealing a higher PJI rate with octenidine. The changes referred to would not be expected to impact on the efficacy of the MSSA decolonisation regime used at the time. As a result of this analysis our organisation returned to using mupirocin as the primary agent. This change was again audited to ensure its effectiveness, 6/72 (8.3%) were positive on admission for surgery. This is consistent with the original decolonisation efficacy suggesting there are no confounding factors that changed over the time period of the study. Over the course of the study the swabs used to acquire samples were changed. Regular internal validation was performed to ensure there was no change in the sensitivity of the techniques and equipment used.

Over time the proportion of patients re-swabbed on admission increased, as such there is a statistically significant difference in the proportion between the groups. This is a potential source of bias. The research team discussed this with the nursing staff responsible for admitting patients, it appears the decision was down to the member of staff’s interpretation of the protocol and not any patient factors.

Due to the low incidence, this study was likely to be underpowered to detect a difference in the secondary outcome, MSSA PJI. A Post-hoc power analysis was performed for our study, for the group 2 versus group 3 MSSA PJI analysis we calculated the power to be 0.27. A sample size of 14,094 would be needed to achieve a power of 0.8.

## Conclusions

There is now good evidence to support MSSA decolonisation therapy prior to major joint arthroplasty and other high risk surgeries. Mupirocin is currently the only agent with strong clinical evidence but mupirocin, neomycin and octenidine are in common use. There is a significant lack of evidence to compare different decolonisation agents. In our non-randomised cohort of 3129 patients we have studied the efficacy of mupirocin, neomycin and octenidine, and found no statistical difference in efficacy between mupirocin and neomycin. However, they were both significantly more effective at MSSA decolonisation than octenidine, with 5.1 times more patients remaining MSSA positive after treatment with octenidine. Any possible differences in the agents and decolonisation did not translate to a difference in deep infection rate.

With the threat of drug resistance further randomised research is needed to establish effective alternatives to mupirocin. The importance of bacterial load must be better understood to develop improved screening and treatment protocols.

## Data Availability

The datasets generated and analysed during the current study are not publicly available due to UK patient confidentiality and data protection legislation.

## Abbreviations

PJI: Peri-prosthetic joint infection
MSSA: Methicillin sensitive *Staphylococcus aureus*
PCR: Polymerase chain reaction
SSI: Surgical site infection
